# New Insights Into Acceptance Across Adulthood

**DOI:** 10.1093/geroni/igaf122.636

**Published:** 2025-12-31

**Authors:** Claudia Haase, Lillian Fu, Chen-Wei Yu, David Rompilla, Michael Kisley

**Affiliations:** Northwestern University, Evanston, Illinois, United States; University of California, Irvine, Evanston, Illinois, United States; Northwestern University, Evanston, Illinois, United States; Texas A&M University, College Station, Texas, United States; University of Colorado Colorado Springs, Colorado Springs, Colorado, United States

## Abstract

Acceptance (i.e., embracing emotions and thoughts without judging them) is an emotion regulation strategy that may become particularly important in late life. While research on other strategies (e.g., positive reappraisal) has been blossoming, we know surprisingly little about acceptance. We present findings from a research program on acceptance that combines laboratory-based and survey-based approaches. Findings from our laboratory-based research show that acceptance of negative emotions (a) is preferred over other strategies (i.e., detachment, positive reappraisal), (b) predicts greater vagal reactivity during instructed emotion regulation (for habitual acceptance), and (c) predicts greater mental health, especially when executive functioning is constrained in older adults (N = 129, age 64-83). In a new line of survey-based research, we examine age differences in acceptance, distinguishing between the acceptance and rejection of positive and negative emotions. Across three studies with age-diverse samples (N = 416, 402, 412; age range: 18-87), findings show that (a) older adults are less likely to reject positive emotions and (albeit less consistently) more likely to accept positive emotions compared to younger adults. Moreover, (b) while no consistent age effects are found for negative emotions broadly, clear age differences emerge for the acceptance and rejection of anger and sadness specifically. Older adults (65+) are less likely than younger adults (18-35) to reject anger and sadness and more likely to accept anger and sadness (with sadness showing significantly steeper age gradients than anger). Together, these findings underscore the important role that acceptance plays in late life and contribute to longstanding discussions about emotion regulation across adulthood.

